# Positive deviance as a novel tool in malaria control and elimination: methodology, qualitative assessment and future potential

**DOI:** 10.1186/s12936-016-1129-5

**Published:** 2016-02-16

**Authors:** Muhammad Shafique, Hannah M. Edwards, Celine Zegers De Beyl, Bou Kheng Thavrin, Myo Min, Arantxa Roca-Feltrer

**Affiliations:** Malaria Consortium Asia, Faculty of Tropical Medicine, Mahidol University, 420/6 Rajavidhi Road, Bangkok, 10400 Thailand; Malaria Consortium, Development House, 56-64 Leonard Street, London, EC2A 4LT UK; Cambodia’s National Centre for Parasitology, Entomology and Malaria Control (CNM), Phnom Penh, Cambodia; Myanmar Medical Association (MMA), No.249, Theinbyu Road, Mingalar Taung Nyunt Tsp, Yangon, Myanmar

**Keywords:** Malaria, Positive deviance (PD), Behaviour change communication (BCC), Elimination, Knowledge, Attitudes and practices (KAP)

## Abstract

**Background:**

Positive deviance (PD) is an asset-based, community-driven approach to behaviour change that has successfully been applied to address many health and social problems. It is yet to have been assessed for malaria control but may represent a promising tool for malaria elimination given its suitability in targeting small and remote population groups, apparent sustainability and ability to instil a high amount of community mobilisation. Here, the PD methodology as applied to malaria is explained, with focus upon and qualitative assessment of a proof of concept study in Cambodia.

**Methods:**

Three villages in Battambang, northwestern Cambodia were selected for the intervention, with an estimated population of 5036 including both residents and migrant workers. In August 2010, field teams conducted a 1 week PD process to sensitise and mobilise the community, establish normative behaviours in relation to malaria control and prevention, identify positive deviant behaviours from within the community, and identify PD volunteers. Until March 2011, PD volunteers were supported by field teams via monthly meetings to conduct activities in their respective communities to increase practice of PD behaviours. In February 2012, 1 year following the end of external support, evaluative interviews were conducted with community members to qualitatively assess community acceptance and interpretation of the PD intervention, perceived behaviour changes, and perceived positive outcomes.

**Results:**

Qualitative data from focus group discussions and in-depth interviews showed that the PD approach was well-accepted into the communities and created a strong sense of community empowerment. Positive behaviour change was linked to the PD intervention, including greater usage of nets by forest goers, and use of public health facilities for malaria diagnosis and treatment. One year following the end of external assistance, PD volunteers were still conducting activities in their respective communities.

**Conclusions:**

PD offers a promising tool in malaria control and elimination settings. Work is ongoing to quantitatively measure impact of PD on behaviours and malaria transmission and once gathered, national malaria control programmes should be encouraged to look at including PD as part of their national strategies. Feasibility of scale-up, cost-effectiveness, and applicability to other settings and diseases is also currently being explored.

**Electronic supplementary material:**

The online version of this article (doi:10.1186/s12936-016-1129-5) contains supplementary material, which is available to authorized users.

## Background

Positive deviance (PD) is an asset-based, problem-solving and community-driven approach to behaviour change that is particularly able to reach high-risk populations [1]. PD was initially conceived in relation to studies on nutrition and has been applied to and assessed in this field in a variety of settings where it has consistently had a marked effect on health outcomes [2–6]. The PD concept has more recently been applied to a variety of health and social research programmes including female genital mutilation, hand washing and hygiene, healthcare performance, maternal and new born health, weight-loss, breast-feeding, retention of antiretroviral patients, reduction of hospital acquired infection and prevention of Chagas disease, among others [7–18].

The general principle of PD is that problems within a community or social group can be better solved by identifying behaviours and practices from within that community that have a positive effect and trying to amplify their use, as opposed to focusing on behaviours that are negative and trying to fix them. The idea is that solutions to most health problems lie within the communities themselves; that there will be a few individuals who deviate from the norm and exhibit unusual but positive behaviours that protect them and their families from certain health problems, despite sharing the same or even more limited resources, having the same socio-economic status (SES), and sharing common risk factors for infection with their neighbours [1]. Identifying individuals from within the communities to act as role models can result in greater engagement from the community and the ability to better-relate to messages than if they were delivered by outside experts or organizations. This latter approach often results in failure because local populations are unable to maintain the practices or behaviours that were identified as lacking once the outside intervention is taken away [19]. Messages and materials from outside experts are often not context-specific or culturally appropriate and, therefore, can lack community acceptance. In contrast, a strong sense of belonging and empowerment can develop from identification of role models with the same resources and challenges—the sense that, “if they can do it, why can’t I.” Positive deviant behaviours are thus affordable, acceptable and sustainable by the people at risk because they are already being practised by others in a similar situation [20]. Furthermore, high community engagement throughout ensures ownership of the process and outcomes, leading to sustainability of the intervention even several years after external influence has been removed [21].

Despite extensive evidence of PD as an effective tool across a range of health-related problems, PD has not yet been fully assessed in the fight against malaria, yet it may offer an effective method of prevention and control, and importantly may be an effective tool in malaria elimination. In the Greater Mekong Subregion (GMS), the burden of malaria is concentrated in high-risk, hard-to-reach groups that are often situated in forested regions, remote border areas or are highly mobile (young adult males who go to the forest are often the most high-risk group in the GMS) [22]. These groups are often difficult to target with classic behaviour change communication (BCC) methods, such as radio/TV adverts or billboards, particularly if they speak a different language or dialect and have different customs. In other instances, communities may have been saturated by currently available tools yet residual malaria transmission continues. In these cases, current tools have reached the limit of their effectiveness and caseloads are low, making it harder to mobilize communities to perform preventive behaviours [23, 24]. Furthermore, there can be a lack of understanding of the specific local context in which people live. The PD process, however, helps to understand their environment and social context so that a culturally appropriate strategy can be defined and implemented based on community role models.

The malaria situation in the GMS has still greater complexity given the failure of artemisinin resistance (AR) containment, with independent emergence of AR, now apparent in many different foci across the GMS [25–27]. This has resulted in a noticeable change in focus of malaria control programmes from one of AR containment, to elimination of *Plasmodium falciparum*, thus requiring the intensive targeting of these remote sub-populations and regions as infection rates decrease and become more concentrated in high-risk groups. To achieve elimination, there is the need for targeted and novel ways to increase malaria prevention practices among high-risk communities. Furthermore, these tools need to be of low cost and sustainable with the inevitable reduction in available funding that comes with successes in control.

PD presents a potential tool to overcome these problems as it is potentially: (i) a low-cost approach that requires few resources, (ii) ideal to target remote and small populations, (iii) sustainable, and (iv) able to create a high amount of community mobilization. According to current knowledge, this is the first application of PD to malaria control. Through conducting a number of small-scale implementation programmes in communities across the GMS, an effective approach has been developed with evidence of real potential for its success in malaria control [28]. Here, the detailed methodology of PD as adapted for malaria control is described along with qualitative feedback from community members following a proof of concept study in Cambodia. This article summarizes the potential of PD as a tool in malaria elimination, as well as current and planned application to other settings and for scale-up of the intervention.

## Methods

The classic methodology for PD has been described elsewhere [20, 29]; here, the PD approach as adapted for malaria is described, with a focus on implementation during a proof of concept study in Cambodia between August 2010 and March 2011. The project evaluation was led by the Cambodia Ministry of Health and all activities, including interviews, were conducted with the full participation of, and were supervised by, senior staff from the Cambodia National Centre for Parasitology, Entomology and Malaria Control (CNM). In line with standard CNM practice it was required that participants provide informed verbal consent prior to interviews. The evaluation was approved by the Ministry of Health of Cambodia and constituted a core programmatic activity of CNM.

### Study site and population

**Fig. 1:**
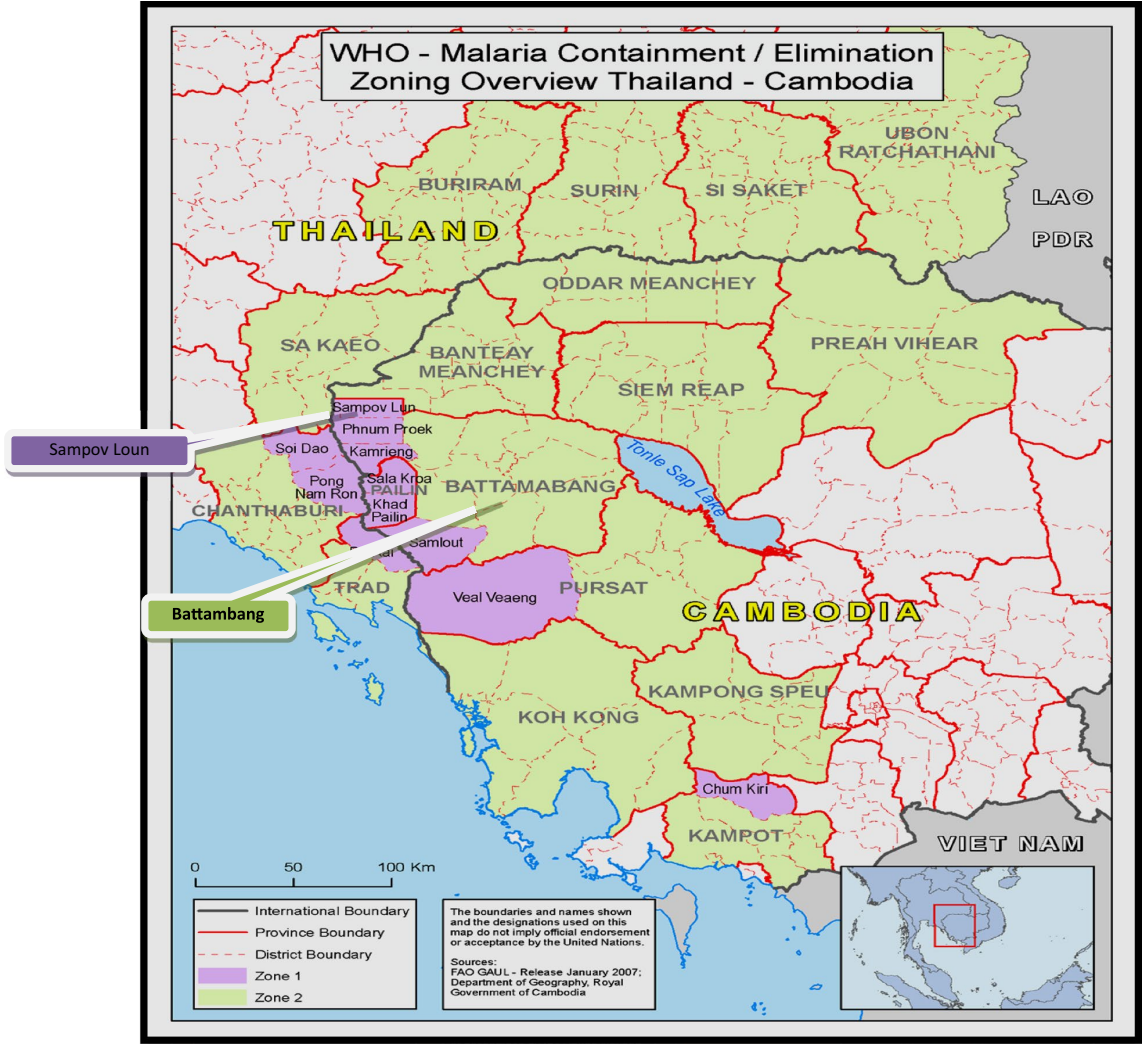
Map of Cambodia displaying province and district of study site

Three villages were selected for PD implementation in Sampov Luon, a sub-district of Battambang province in north-western Cambodia (Fig. 1). The villages of Kampong Chamlang Leu, with a population of 759 residents and 550 migrants; Ploav Praim Muy, with 1402 residents and 869 migrants; and Samsep, with 1072 residents and 384 migrants (figures obtained from personal communication). The total population of 5036 individuals thus included both residents and migrant workers. It is traditionally an area of farming, with crops such as corn, cassava and rice.

Malaria incidence in 2010 was 0.2 per 1000 population in Kampong Chamlang Leu, and 0.02 per 1000 population in Samsep; there were zero cases reported from Ploav Praim Muy. The area was classified as Zone 1 under the AR containment project, meaning it is at highest risk of AR transmission. The villages were selected due to their classification as high-risk Tier 1 villages under the artemisinin resistance containment project. Tier 1 areas are areas that had documented proof of artemisinin resistance. Villages were also selected that had an influx of mobile and migrant populations (MMPs) and because they shared the same health centre catchment area. The idea of having a shared heath centre was to provide a base for upcoming activities and to link the PD intervention with the permanent health system for greater chance of sustainability once external help had been removed.

### The positive deviance protocol

The PD intervention can be split into two phases: the PD Process, and the implementation period, with the latter ending in a handover of the project to the community. Table 1 summarizes the key processes and outputs of the methodology. In Cambodia, the PD process took place over 1 week in August 2010. Subsequent implementation activities were conducted up to February 2011, with a community handover seminar in March 2011.

**Table 1:** Summary of key processes and outputs in the PD methodology

	Key outputs
Key activities in the PD process	
1. Key stakeholder meeting	Inform key figures and gain support for project Map community groups
2. Community orientation meeting	Community sensitisation and mobilisation Identification of participants for FGDs and IDIs
3. Situation analysis	Understanding of context and normative behaviours related to malaria
4. PD inquiry	Identification of PD behaviours and role models
5. Participatory analysis	Triangulate findings with key stakeholders
6. Community feedback session	Share key PD behaviours Recruit PD volunteers Action plan for PD activities
Key activities in the PD implementation process	
1. Training of PD volunteers	PD volunteers understanding of malaria and PD behaviours; strategies for communicating to community; action plan for activities
2. PD interactive sessions by volunteers	Spread PD messages to different community groups
3. Monthly volunteer meetings	Feedback and action planning
4. Monitoring of malaria cases and PD activities	Village malaria maps PD activity coverage maps
5. Community handover seminar	Sustainability of intervention by community

### The PD process

The PD process involved a 1 week set of activities to mobilise and sensitise the communities to malaria prevention and control, understand normative behaviours around malaria prevention and control within the community, and discover uncommon beneficial behaviours and strategies already being practiced by some community members. The PD process ended in defining a strategy for implementing activities within the community that can increase practice of these behaviours.

The PD process was conducted in a series of six stages over the course of one week under the leadership and supervision of an experienced behaviour change, community mobilization and qualitative research specialist, with a team of experienced qualitative interviewers, fluent in the local language and with experience in community mobilization activities:

#### 1. Pre-orientation meeting

A meeting was held with key stakeholders from the target communities, as well as district and provincial health department and national malaria control programme (NMCP) staff. Key stakeholders included, community health workers/village malaria workers (VMWs), Health Centre staff, NMCP staff, and Village Chiefs. The main purpose was to inform the community leaders and gatekeepers of the project to ensure their support in the upcoming activities.

Once oriented and support to the project had been granted, a map of each target village was created with the respective village chiefs and health volunteers to understand the demographics of the different sub-populations living in different parts of the village, i.e. different migrant groups, ethnic minorities, and those with a different SES. This was to ensure equal representation from each sub-population at the PD process meetings and activities.

#### 2. Community orientation meeting

**Fig. 2:**
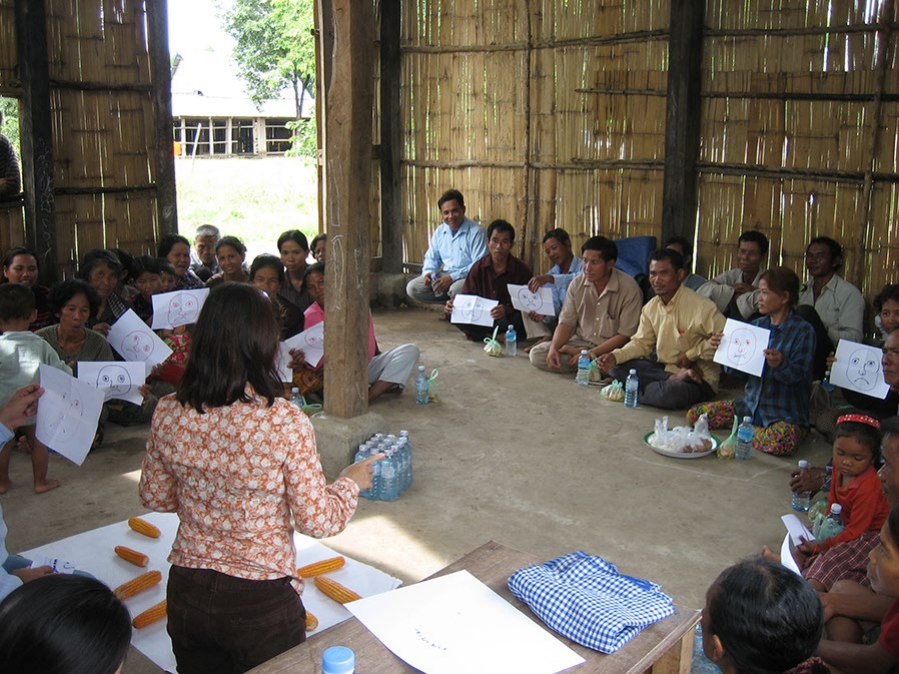
Conceptual game played during community orientation meeting

Equal numbers of individuals from each identified sub-population within the target villages (around eight–ten individuals per group) were invited to attend an orientation meeting. Participants included community residents, migrant workers, teachers, religious leaders, health volunteers and health facility staff. The main purpose of the meeting was to introduce the PD concept through different interactive activities. The field team discussed with the community members and sensitized them to the malaria problem. Explanation was given, with the help of different conceptual games, about how the PD approach helps to identify solutions from within the community (Fig. 2 and Additional file 1). Throughout this stage, the field team identified interested volunteers to participate in the next stage, the situation analysis.

#### 3. Situation analysis

**Fig. 3:**
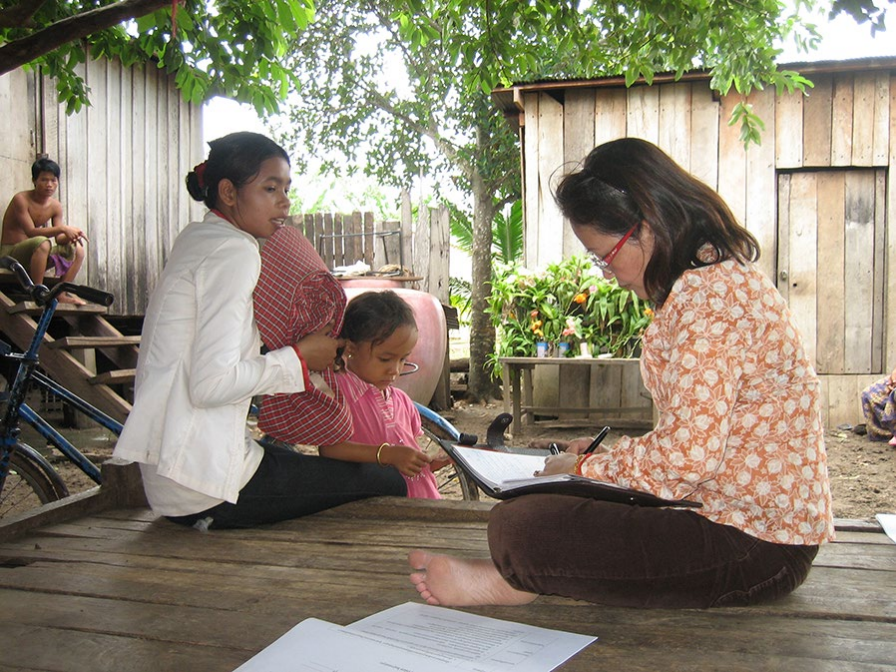
An IDI being held with a community member. Purposive and convenience sampling was used to ensure IDIs were held with individuals from different groups within the communities and to ensure gender balance

Focus group discussions (FGDs) and in-depth interviews (IDIs) were conducted with the help of selected volunteers in each target community to establish the normative behaviours around malaria prevention and control in the communities (Fig. 3). The volunteers simply helped to organize the FGDs and IDIs by orienting the community members and assisting to provide feedback after the interviews. Bed nets and hammock nets were used to animate the discussions and ensure precise and high quality information in regard to the use of nets.

Adult men and women aged 18 years and above were targeted for the FGDs and IDIs, and equal numbers of male and female participants were sought in order to have a fair account of the perspectives of both sexes. IDIs and FGDs were conducted with members from each sub-population within the community, including mobile and migrant workers, landlords, and community residents. Purposive and convenience sampling techniques were used to collect information from key respondents.

During the IDIs and FGDs, comprehensive notes were taken by two experienced note-takers. All IDIs and FGDs were also audio-recorded. The field notes were transcribed verbatim and translated into English the same evening to reduce recall bias. Questions asked in the IDIs centred on themes of bed net use, healthcare-seeking behaviour, treatment adherence, and work routines. In FGDs, field teams prompted discussion on the signs and symptoms of malaria, causes of malaria, healthcare-seeking behaviour, preventive measures, communication channels, migration patterns and work routines.

Responses from the IDIs and FGDs were grouped under main topics and themes used in data collection. The data was triangulated using information from IDIs and FGDs with other respondents, by conducting a transect walk through the villages to observe context and behaviours (by the trained project leaders and implementers), and were reviewed and validated with the field team involved in the data collection.

#### 4. Positive deviance inquiry

The PD Inquiry was to identify individuals within the community that practice uncommon positive behaviours related to malaria prevention and control. Classic PD methodology as used in childhood nutrition studies, for example, involved project teams weighing children or pregnant women to identify those with healthier weight [2–6], and then interviewing their families to find out what behaviours were being conducted that were out of the norm.

Identifying positive deviants in malaria required a different approach because it is not immediately apparent who avoids contracting the disease. Thus, the methodology for malaria PD required two layers of in-depth probing. Firstly, FGDs and IDIs with key respondents in the situation analysis were used to identify individuals from the community (i) that had never had malaria, and thus may represent positive deviants in relation to prevention practices, and (ii) that had had malaria in the past but performed different healthcare seeking behaviours to the rest of the community.

A second stage of interviewing was then conducted with these identified individuals to discover which of them practised uncommon positive behaviours, and thus that could be demonstrated as PD ‘role models’. Upon discovery of uncommon positive behaviours, in-depth probing was used to understand the determinants that enabled execution of these behaviours and strategies. The probing questions were designed to understand the enabling environment at the household or community level, challenges that are faced and how these challenges are overcome. PD role models were selected as such only if they did not have access to special resources, such as the husband/wife of a healthcare provider, or village chief (rich person), that increased their ability to carry out the behaviours.

Answers were recorded and analysed in the same way as the situation analysis.

#### 5. Participatory analysis

**Fig. 4:**
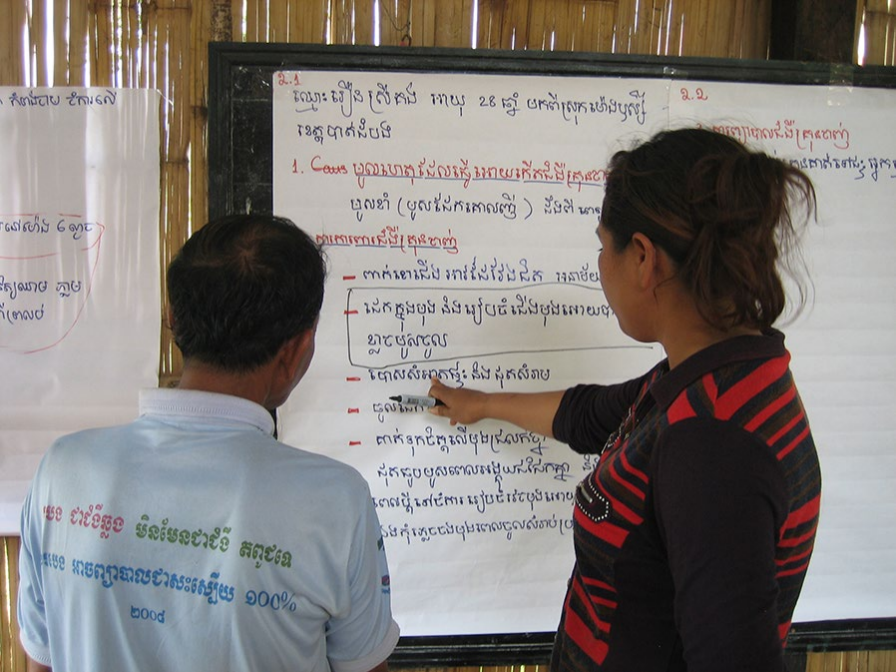
Participatory analysis. Project staff and community members participated in joint analysis of the data from IDIs and FGDs to ensure validation of findings and community participation

This stage was a new addition to the classic PD methodology in order to increase the level of community engagement and participation, as well as validate findings from the interviews. Following the FGDs and IDIs, a meeting was called of key community stakeholders. Field teams displayed all positive behaviours reported by the participants during the discussions and interviews on paper on the side of a wall in a community building. Community stakeholders were asked to read through, discuss and validate the responses, and identify those that were uncommon, and thus that were deviant behaviours (Fig. 4).

#### 6. Community feedback session and action planning

**Fig. 5:**
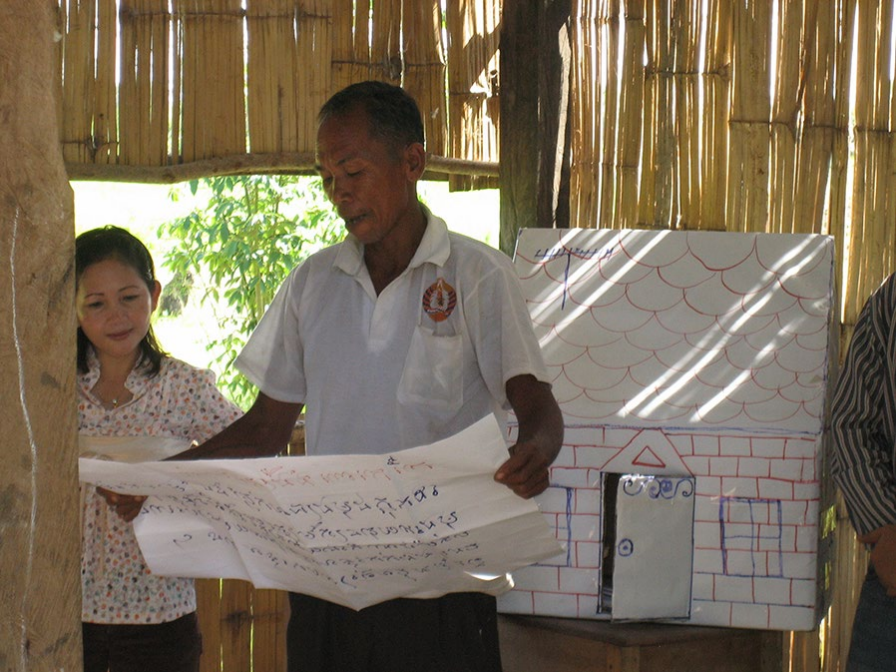
Sharing positive behaviours with the community

Once the PD behaviours had been identified, community members who took part in the community orientation meeting were invited to receive feedback (Fig. 5 and Additional file 1). Original participants ended up being accompanied by other interested community members, increasing the number of people involved. The PD process was reviewed, findings were shared and activities/interactive games were conducted to mobilize and motivate the residents, migrants and landlords to devise strategies to enable other community members to adopt these behaviours.

### PD implementation period

Following the PD Process, a period of implementation led by the field team was conducted. This period ended with a symbolic handover of the project to the community for them to continue and sustain the project by themselves. The implementation period had the following activities:

#### Identification and training of volunteers

As a new concept to the classic PD approach, individuals from the communities that practised positive deviant behaviours were recruited to be PD volunteers. Community health volunteers were already present in the community, but the idea was that the PD volunteers would add value because they were chosen to be representative of groups within the community (e.g. same SES and availability of resources). They could work alongside the existing volunteers to share their own personal and inspiring stories to community members, leading to a greater sense of community empowerment. The PD volunteers were linked to the nearest health centre to strengthen linkages between the intervention and the health system and attempt to ensure greater sustainability of the intervention. To ensure inclusivity, anyone that requested to be a volunteer was given opportunity to be one. In the Cambodia setting, a total of 10 PD volunteers were recruited to be part of the intervention, three of whom were PD role models identified from the PD process and the rest community members (including migrant workers, land owners and village residents) eager to be part of the activities. All were entirely voluntary as no incentive was paid besides reimbursing travel costs for monthly meetings. Village health volunteers already working in the villages under the training and supervision of CNM were also engaged in the project.

A two-day training was conducted covering key competencies, such as communication and facilitation, roles and responsibilities, and malaria prevention and control. The main focus was on the positive deviant behaviours and practices that had previously been identified. Interactive and participatory techniques, such as brainstorming, group discussions with visual aids, role plays and games, were used in the training sessions. At the end of the training sessions, volunteers made a work plan to conduct PD activities in their communities.

#### PD interactive sessions

Regular PD sessions were carried out by the PD volunteers in their target communities to share positive deviant practices. Each month, the PD volunteers conducted two sessions to which they invited 20 participants, of mixed gender and from both resident and migrant groups. These sessions were organised in the evenings to maximise opportunity for male forest-goers to attend. Activities included role plays, and sharing of stories and examples of PD from specific people within the community. Additionally, PD volunteers each visited 15-20 households per month to share PD behaviours and reinforce messages from the group sessions.

#### Monthly meetings

Monthly meetings between PD volunteers and project coordinators were held at the local health facility to discuss and share problems and achievements using the “triple A process”—assessment of situation, analysis of problems and action/solutions [30]. A small training session on communication skills or malaria was conducted in each meeting to refresh volunteers’ knowledge. A work plan was developed with the volunteers for the next month and regular feedback was provided to the volunteers on their activities.

#### Monitoring

**Fig. 6:**
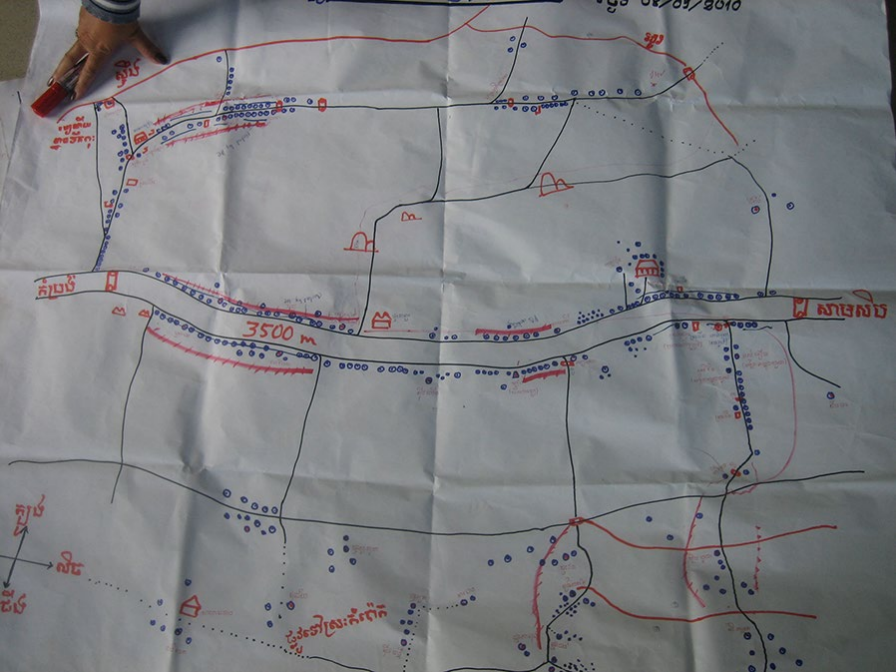
Village malaria map created by PD volunteers. PD volunteers mapped houses in the village, the coverage of PD activities, and houses with confirmed and suspect malaria cases

A further innovation in the PD process for malaria, was development of “village malaria maps” (Fig. 6) by the PD volunteers in each village to record malaria cases and show the coverage of PD sessions, including number of participants in each PD session (disaggregated by gender). Houses were highlighted in different colours to distinguish those that had had fever (considered as suspect malaria) and those that had had confirmed malaria cases. The malaria cases marked by the volunteers were confirmed by the health facility workers during these monthly meetings. The households were also divided into different sections to ensure coverage of PD activities across all sections. The village maps were updated on a monthly basis and presented in the monthly meetings to assess the situation and plan upcoming activities, particularly to cover any gaps in coverage of community members or to target those with suspect/confirmed malaria.

#### Community handover seminar

**Fig. 7:**
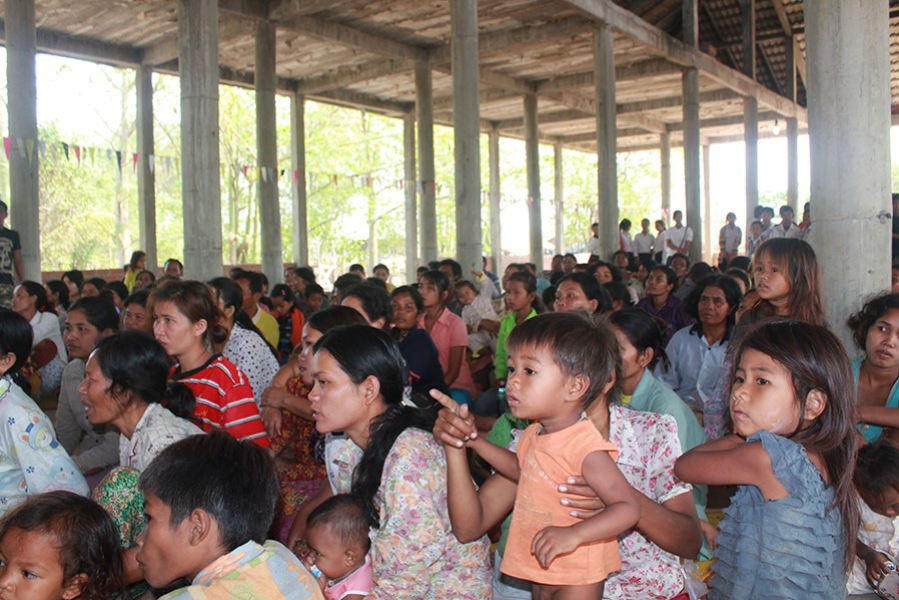
Community handover seminar. A symbolic handover of the project to the community to encourage them to continue the intervention without external help

At the end of the six-month implementation period, a community event was held to officially handover the project to the community (Fig. 7). The main objectives of the seminar were to reinforce key PD messages; encourage and acknowledge the PD volunteers; and handover the PD project to the target communities to ensure sustainability.

The PD seminar was attended by the majority of community members from target PD communities, as well as representatives from the local health facility. The community was involved in the preparations prior to the seminar and various community-based competitions were organized to increase participation at the grass-roots level (Additional file 1). The seminar ended with a symbolic handover of the project to the community for them to sustain by themselves.

The handover seminar was a new concept in the PD approach and it was added in the interests of increasing community empowerment and potential sustainability of the intervention once external help had been removed.

### Data collection

The project field team comprised of four members, each with experience in community mobilization and qualitative methods. A one day training was sufficient to refresh their interviewing techniques, data collection and handling methods, informed consent and research ethics knowledge. The field team led the PD Process and interviews, and field coordinators performed routine monitoring visits to the communities and led monthly meetings with volunteers. Topic guides to be used in interviews were initially developed in English. After discussions with the field team, guides were modified and translated into the local language.

### Qualitative evaluation of Cambodia implementation project

Following the community handover in March 2011, the field team returned 1 year later in February 2012 to conduct a qualitative evaluation of the PD process and short-term sustainability of the project. FGDs and IDIs were conducted using the same methodology as in the situation analysis and PD Inquiry. Questions and topics of discussion in the evaluation centred on the acceptance and interpretation of the PD intervention, perceived changes in behaviour of the community members and any associated outcomes or benefits from the project.

## Results

### PD process

In total, 13 IDIs (six with mobile and migrant workers, two with landowners, and five with residents and village leaders) and six FGDs [three with mobile and migrant workers (two male, one female) one with landowners (male) and two with community residents/village leaders (one male, one female)], each with eight participants, were conducted as part of the situation analysis and PD Inquiry. A summary of the normative behaviours identified from the three villages is described in Table 2. Following the participatory analysis, PD behaviours were recognised from the community as described in Table 3. A total of ten PD volunteers were recruited to be part of the intervention, three of these were the PD role models and the remainder were volunteers eager to be part of the activities.

**Table 2:** Main normative behaviours and attitudes reported during the PD situation analysis in Sampov Luon, Cambodia, 2010

Migration	Knowledge of malaria	Healthcare seeking behaviour	Preventive behaviours
Majority of migrant workers come for seasonal work in the area Majority stay 15–30 days before returning to their hometown A few migrants stay up to 3–4 months in the village Both male and female migrants are hired by landowners Migrants stay with the landlords in the village	Most respondents correctly identified signs and symptoms of malaria Most respondents mentioned that mosquitoes spread malaria, but many related it to other causes such as dirty water, unhygienic environment, forest spirits and ‘changing of the land’ Men, especially male migrant workers, were recognised to be the highest risk group due to conducting activities in the forest and being out at night working or socialising	Delay in seeking healthcare for 1–2 days was a norm upon presentation of fever Most respondents reported that they first consulted private drug sellers for treatment, and only if symptoms persisted would they go to health centre for diagnosis Many reported using traditional medicine or coin rubbing when they get fever Many preferred public facilities due to low cost; however, landowners often preferred private sector due to distance to heath centre and unreliable availability of staff or medicines at health centre Many visited VMWs for testing but stated frustration that VMWs cannot do anything with a negative test result	Ownership and usage of LLINs by residents was high Many residents reported wearing long sleeves and burning coils Ownership and use of LLINs or LLIHNs by migrants was low Reports of holes in LLINs was common Correct usage of bed nets was poor Some landowners kept extra nets to give to their migrant workers

*LLIHN* long-lasting insecticide-treated hammock net, *LLIN* long-lasting insecticide-treated net, *VMW* village malaria worker

**Table 3:** PD behaviours identified during the PD process in Sampov Luon, Cambodia, and their link to strengthening other standard control initiatives

	PD behaviour	Link to other control strategies
Bed net use	Correct usage of bed nets—clearing mosquitoes from the net first, tucking into mattress, repairing holes, etc	Universal coverage has been achieved in many areas of the GMS, yet residual transmission remains. Usage of bed nets and correct usage is a key factor in achieving maximal impact from universal coverage
	Usage of LLIN/LLIHN among migrant community and by forest-goers	Transmission in the GMS is characterized by forest transmission and high-risk in MMPs, who are difficult to target with malaria control initiatives
	Landowner that keeps extra nets to supply to migrant workers	There is difficulty in targeting MMPs for malaria interventions and commodities distribution. Landowners represent a potential target point of access
Healthcare seeking	Seek treatment for fever without delay	Delay in seeking treatment contributes to malaria transmission
	Seek diagnosis and treatment from public health facilities or VMWs	In Cambodia, cases at PPs are mostly not reported to the national surveillance system. Use of facilities where cases are linked to surveillance systems is critical in pre-elimination and elimination settings
	Landowner that encourages migrant workers to go to the health centre if sick	There is lower uptake of services by MMPs. Landowners represent a potential target point of access
General	Cover arms and legs in the evening	Prevention of mosquito biting to lower transmission

*GMS* greater Mekong subregion, *LLIN* long-lasting insecticide-treated net, *LLIHN* long-lasting insecticide-treated hammock net, *MMP* mobile and migrant population, *PP* private provider, *VMW* village malaria worker

### Qualitative evaluation

A total of five IDIs (two with VHVs, one with the Health Centre Chief, two with Village Chiefs) and nine FGDs [four with migrant workers (two male, two female) and five with residents (two male and three female)], each with eight participants, were carried out as part of the evaluation survey in February 2012. Responses were grouped into three common themes: community interpretation of the PD intervention, behavioural changes, and household and community level outcomes.

### Community interpretation of the PD intervention

The majority of community members were aware of the PD project and it was acknowledged to be a useful and empowering approach to identify and practice the positive behaviours of PD role models from within the same community.

*“The interesting thing was that we identified the PD role models from the same community. We adopted their behaviours which were very easy to follow. We feel proud that these are our own people doing something positive and different.” FGD*—*male community member.*

*“After learning from the PD role model, we follow her behaviours. We wear long sleeved clothes and sleep under the bed net every night to protect ourselves from malaria.” FGD*—*female community member.*

Most community members and migrant workers also mentioned that they participated in and enjoyed the PD project activities, particularly role plays during monthly PD sessions and the community seminar.

*“We regularly participated in the PD sessions that were very interesting and different from the routine health education sessions. The real PD individuals shared their personal experiences and practices which were very useful and simple to practice.” FGD*—*female community member.*

*“The community members really enjoyed the colourful event of the community seminar. They enjoyed the song and malaria poster competitions. We never witnessed such interesting activities before this project.” IDI*—*PD volunteer.*

Respondents noted that the PD process was a catalyst for change because it opened their eyes to behaviours being conducted within the community, from their own people. By identifying these people, the community had “social proof” that they could also conduct these behaviours.

*“The best thing is that PD role model belongs to the same community. People can personally relate with the PD role model individuals and feel proud that they are their own people practicing positive/uncommon behaviours.” IDI*—*Health Centre Chief.*

Importantly, respondents reported that PD initiatives were still continuing within the community 1 year after the symbolic handover of the project by the external field team.

*“Even after the PD project finished a year ago, the PD activities i.e., PD sessions are still continued and we meet regularly in bi*-*monthly meetings with the chief of Takrey health centre to plan and conduct the PD activities.” IDI*—*PD volunteer.*

*“The PD project has increased knowledge and improved the behaviours of the community members and migrants regarding malaria. We want to continue this project to bring further improvements in our knowledge and behaviours.” IDI*—*Village Chief.*

### Behavioural changes

It was reported that there were clear positive changes in behaviour among the resident community and the migrant population, including wearing long-sleeved clothes in the evening and in the forest. Although it had been high among residents prior to the intervention, it was reported that bed net use had further increased and that more people were using their bed nets correctly.

*“Now community members wear long sleeved shirts in the evening to prevent mosquito bites as demonstrated by one of the PD role model”. IDI*—*village health volunteer.*

*“Another change regarding the use of bed net is that before when our nets were torn off, we never mend or patched them. However, after learning from the PD role models, we use insecticide treated bed nets every day and if a net is torn off or has a hole, we mend it as soon as possible. However, if the whole is too big, we buy a new net”. FGD*—*female community member.*

As was the case during the PD process, the majority of community members and migrant workers mentioned that forests are high-risk areas, and that men are more at risk of malaria. However, during FGDs in the evaluation, most of the migrant workers mentioned that malaria among forest-goers had reduced because they now use long-lasting insecticide-treated hammock nets (LLIHNs) and wear long-sleeved clothes in the forest to avoid malaria, both key messages from the PD role models.

Prior to the PD implementation, most community members had reported using private drug shops or pharmacies to receive malaria treatment, however, during the evaluation interviews, the majority of people mentioned that they now go to the health centre or VMW for a blood test and malaria treatment if they have fever.

*“After learning from the PD role model…whenever we have fever we do not go to drug shop, we straight away go to health centre for the treatment.” FGD*—*male community member.*

*“Whenever we get sick and have fever, we go to VMW for the diagnosis or treatment. Our landlord also suggests us to go to VMWs for treatment.” FGD*—*female migrant worker.*

Despite this, many respondents mentioned that they still wait 1–2 days for symptoms to develop before they visit the health centre for treatment, citing lack of money, lack of transport, and poor road conditions as delaying factors. Some also mentioned that they still use traditional medicine first and if not cured then they go to the health centre for treatment.

### Household and community level outcomes

Community members and migrant workers suggested that the following changes had occurred due to the PD intervention:

#### Malaria cases decreased

The chief of the health centre reported that the number of malaria cases within the catchment area had decreased from 48 cases in 2010, to 24 cases in 2011. Only six cases of malaria were found from the three PD villages over the last year, which he attributed to the PD intervention. The reduction in malaria cases was also associated with an improved financial situation among community members.

*“We did not get malaria this year which enabled us to save some money which we otherwise used to spend on malaria treatment. We can spend this money to fulfil our other important needs.” FGD*—*male community member.*

*“The PD project has helped reduce malaria among community members and migrant workers and hence reduced poverty.” IDI*—*Village Chief.*

#### Malaria knowledge increased

Despite knowledge of malaria signs and symptoms being high prior to the intervention, it was noted that the knowledge among the PD communities had increased. It was also stated that the VHVs of PD villages were more confident, motivated and skilful than volunteers from other villages. However, the cause of malaria was still attributed to reasons other than mosquito bites, including ingestion of un-clean water, unhygienic food, changing of land and forest spirits.

## Discussion

PD has tremendous promise as an effective community mobilization and behaviour change tool in malaria control and elimination settings. Here, community feedback following a small-scale evaluation in selected villages of Cambodia has been presented; however, the approach has since been applied to sites in Myanmar and Thailand and similar feedback from community members has been received.

Instilling confidence in the applicability of this intervention across different settings. In all areas the PD activity was well-accepted into the community and created a high amount of motivation and empowerment among community members to perform the PD behaviours identified, which in turn was associated with positive outcomes of behaviour change and fewer malaria episodes. Of real promise, is the apparent sustainability of the intervention, with activities still performed in the target communities in Cambodia 1 year following the community handover seminar.

### Suitability to malaria control and elimination

PD is particularly suited to targeting small, remote communities, which are a prominent feature of malaria risk in the GMS [22], and to other areas reaching malaria pre-elimination and elimination status. Classic BCC interventions using media outlets, such as TV and radio, often cannot reach these communities due to poor electricity supply, network coverage, or the fact that those at risk are often out of doors for long periods. BCC messages are often not context-specific and communities can reach saturation point if constantly bombarded with them. Classic BCC approaches often follow a needs-based approach which means communities may be unable to find a way to meet and sustain the behaviour [19]. PD overcomes these challenges by involving the community from the beginning and throughout the whole process, learning from them about their day-to-day environment and challenges faced, and identifying role model behaviours from within the community.

Maintenance of community participation and enthusiasm in times of diminishing disease becomes a great challenge, and practice of preventive behaviours can fall because there are very few or no cases and people forget how important/serious the disease can be; it is no longer considered a threat [24]. The PD approach presented here was well accepted by the target communities, with high community engagement and motivation to participate throughout. The interactive nature of the activities and identification of role models from within each group in the community created a strong sense of empowerment among all community members and eagerness to take part in activities. It was easy to recruit volunteers to conduct the PD community sessions and individuals reported that they could more easily relate to the role models and behaviours they needed to follow. This sense of community cohesion should be a positive influence on uptake of behaviours even when number of cases is low. Thus, PD represents a strong community mobilisation tool that engages communities well even in elimination contexts.

In elimination settings, it is also highly important to find interventions that are sustainable over a prolonged period of time. Where countries have had successes in malaria control and reduced numbers of cases, there follows an inevitable refocus of donor interests to countries with higher burden of disease, resulting in less money and fewer resources available to sustain malaria control activities [31]. Control and surveillance methods required in elimination stages are also often costly [32]. Although resource intensive at the beginning, PD has the potential to be sustainable at very low cost (see below for limitations and challenges). High community engagement and empowerment, along with the symbolic handover of the project may well ensure sustainability of the intervention after activities by the project field staff have ended. Evidence of short-term sustainability was found in Cambodia when PD volunteers were still conducting activities 1 year after external support from our team had stopped. Investigation needs to be done to determine the sustainability over a longer time-frame and whether top-up sessions with the community or PD volunteer retraining would be of benefit, and at which point in time it would be required.

Furthermore, PD has the potential to foster other interventions such as participatory surveillance [33], as demonstrated through the development of community malaria maps, which will become particularly critical in the last push to malaria elimination.

### Limitations and challenges

A key limitation in advocating for the use of PD in malaria control is the lack of quantitative evidence that it has an impact on human behaviour, and a subsequent impact on malaria transmission and disease. Here, we report qualitative information from community feedback that there was behaviour change in the community, and respondents in turn associated this with a reduction in malaria cases; however, this was not tested and the drop in cases may be attributable to a variety of other factors, such as low rainfall, impact of other interventions, etc. Behaviour change is notoriously difficult to measure, but attempts can be made to quantify this through surveys and observational studies. However, behaviour change in itself is not the ultimate goal; behaviour change is only useful if it has an ultimate impact on malaria transmission. Thus, the use of epidemiological indicators to assess PD, such as malaria incidence and prevalence rates, needs to be explored. Challenges with the prospect of using behaviour change and epidemiological indicators are firstly, that the populations in the GMS are generally highly mobile, so may differ between baseline and end-line surveys; and secondly, that positive behaviours in these communities are already very common and malaria caseloads are already very low, so massive sample sizes are required in order to detect any statistical difference. An alternative may be the use of entomological indicators. Use of entomological indicators is already being explored for the assessment of PD in dengue control, through measurement of pupal and larval density indices, and this could also offer a potential quantitative method of assessing PD in malaria.

Efforts also need to be made to examine the cost-effectiveness of the PD intervention. The initial (but short) PD process is time and labour-intensive and requires individuals with some experience in qualitative research and data collection, and with community mobilization skills. There is also the necessity for high quality supportive supervision of volunteers during the initial implementation phase. However, should the intervention be successfully sustained by the community once external help has been removed, the long-term running of the intervention would essentially be cost-less. Short-term (1 year) sustainability was shown by the intervention in Cambodia. Longer-term sustainability needs to be investigated, along with a comprehensive cost-effectiveness analysis to confirm whether the intervention is indeed low-cost and high-impact compared to other community mobilization and behaviour change interventions. Efforts are now being made to assess this, as well as feasibility and costs of scale-up to a national level.

In areas that have had intensive malaria control activities, it may be difficult to identify positive deviant behaviours because the communities will already have high knowledge of malaria and use of preventive behaviours. In pre-elimination and elimination settings, universal coverage of nets would have been achieved and communities would have received multiple BCC initiatives to result in high practice of preventive behaviours. However, evidence from the GMS shows that even in areas where this has occurred, there remains some residual transmission [34]. In this case, when other control interventions have been exhausted, it is important to find small deficits in prevention practices that can make any difference toward lowering residual malaria transmission. In the Cambodia context, community residents had a high amount of bed net use, however, by using nets to animate discussions, it became clear that there was a deficit in the correct usage of nets, e.g. to clear mosquitoes from the net, ensure it is tucked into the mattress/blanket, and to fix holes. Thus, although difficult, it was still possible to identify deviant behaviours within the communities that would not have been immediately apparent from use of standard surveys [35].

Some aspects of knowledge and behaviour will still be a challenge to address, for example, some of the beliefs around the causes of malaria transmission and treatments for fever are based on cultural and spiritual beliefs and will be very hard to change; while others also rely on external factors, such as having enough money to travel to the health centre.

There are also challenges in targeting the most high-risk sub-populations within the target communities, usually males, forest workers, and MMPs [22]. It was noted during our PD projects that males participated in the PD activities less frequently than females, because they were often at work or in the forest away from the village when PD activities are likely to take place. PD volunteers should encourage community members to cascade messages received at PD sessions through their families and social networks to ensure coverage of all community members.

Population movement in the GMS is highly complex, with a mix of short-term and long-term migrants, high internal mobility as well as international, and frequent border crossing; presenting a challenge for targeting them for PD [36]. During the PD pilot in Cambodia, for example, the target villages had a consistent influx and efflux of migrant workers who usually only stay for up to 30 days, and a maximum of up to 3–4 months. New migrants may not have been exposed to the initial PD Process nor have been involved in any of the monthly PD activities held by the volunteers. For PD to be effective in target communities, regular PD activities need to be sustained and targeted to newly-arrived mobile and migrant individuals and families.

### Future application of PD

Despite these challenges, the evidence provided here, the appropriateness of PD for malaria control and the recorded impact of PD on other health-related issues gives ample hope that the intervention can be successfully applied to malaria control and elimination programmes.

Following the initial proof of concept studies, better ways to assess the impact of PD are being explored, through quantifying behaviour change and measuring epidemiological and entomological indicators in target communities. This will give solid evidence of the impact that PD can have and will inform national malaria strategies of the effectiveness of PD as an elimination tool. A study into the use of PD for dengue control is underway in Myanmar, using entomological end points as a measure of impact, through measurement of pupal and larval density indices, which will gather evidence on the feasibility of these indicators. Furthermore, possible integration of PD for malaria, dengue, and other diseases and health issues should be considered, and it is essential to test the application of PD to target communities using points of access other than villages to maximize coverage of high-risk populations, such as at rubber plantations, farms, or other areas of migrant employment.

Next steps also include exploring the scaling-up and cost-effectiveness of PD at national level, as well as looking into the long-term sustainability of the intervention in target communities. In various national and regional meetings since this implementation, CNM and other NMCPs have showed their interest and commitment to use PD as an effective tool to reach out to high risk and hard-to-reach populations, particularly in elimination settings. As such, the scalability of PD and ability to integrate into national programmes is now being tested.

## Additional file

10.1186/s12936-016-1129-5 Supplementary information regarding the PD process and implementation activities. More in-depth description of the types of community engagement activities that worked well during the PD process in Cambodia.

## Abbreviations

AR: artemisinin resistance
BCC: behaviour change communication
CNM: National Centre for Parasitology, Entomology and Malaria Control
FGD: focus group discussion
IDI: in-depth interview
GMS: greater Mekong subregion
KAP: knowledge, attitudes and practices
LLIHN: long-lasting insecticide-treated hammock net
LLIN: long-lasting insecticide-treated net
MMP: mobile and migrant population
NMCP: National Malaria Control Programme
PD: positive deviance
PP: private provider
SES: socio-economic status
VHV: village health volunteer
VMW: village malaria worker
