# Supplementary material for: Positive deviance as a novel tool in malaria control and elimination: methodology, qualitative assessment and future potential
Source: Malar J. 2016 Feb 16;15:91. doi: 10.1186/s12936-016-1129-5 (PMC4754848; doi:10.1186/s12936-016-1129-5)
Supplement: Supplementary file 1 — 10.1186/s12936-016-1129-5 Supplementary information regarding the PD process and implementation activities. More in-depth description of the types of community engagement activities that worked well during the PD process in Cambodia. [file 12936_2016_1129_MOESM1_ESM.docx]

**Supplementary information regarding the PD process and implementation activities**

More information about the types of activities conducted in various parts of the PD process and implementation period is given below:

1) Community orientation meeting

Various conceptual games were played with the key community stakeholders. In one game, community members were requested to bring 10 pieces of corn or dirt-clod (something that was locally available) and place them onto a large piece of paper (Figure 2). The corn represented the households and the paper represented their village. Beneath the corn the facilitators placed sketches of two happy and eight sad faces. The happy faces represented healthy people and the sad faces represented people who were sick from malaria. One-by-one, community members lifted up each “household” and gradually ascertained and discussed that the healthy outcome results from “good practices” within the family.

2) Community feedback session

Activities conducted included a role play, whereby couples from the audience acted out some of the PD behaviours for the other community members. Community members frequently reported that they enjoyed the role plays and promised to follow the behaviours highlighted.


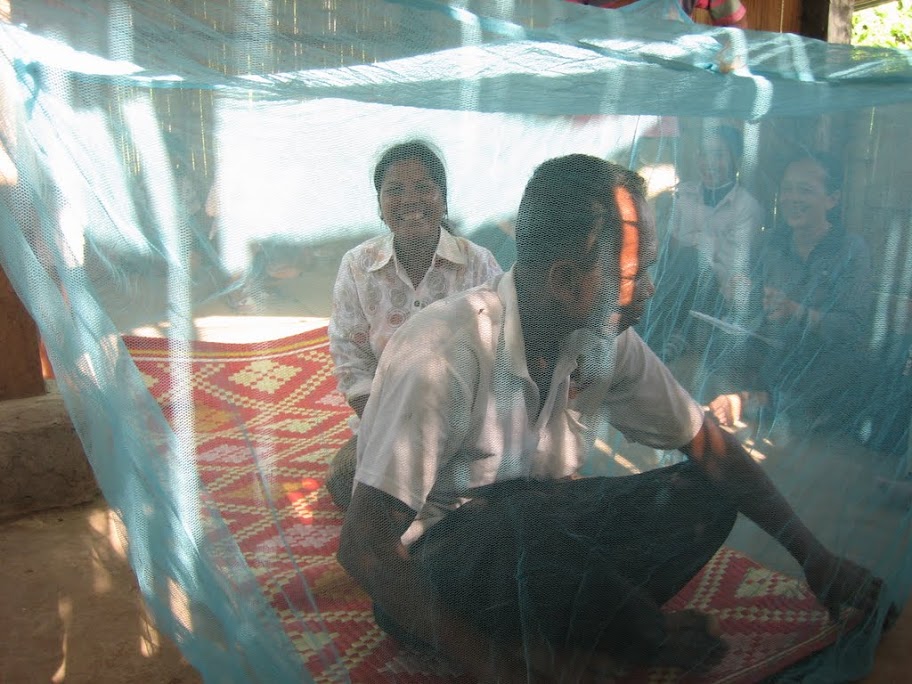


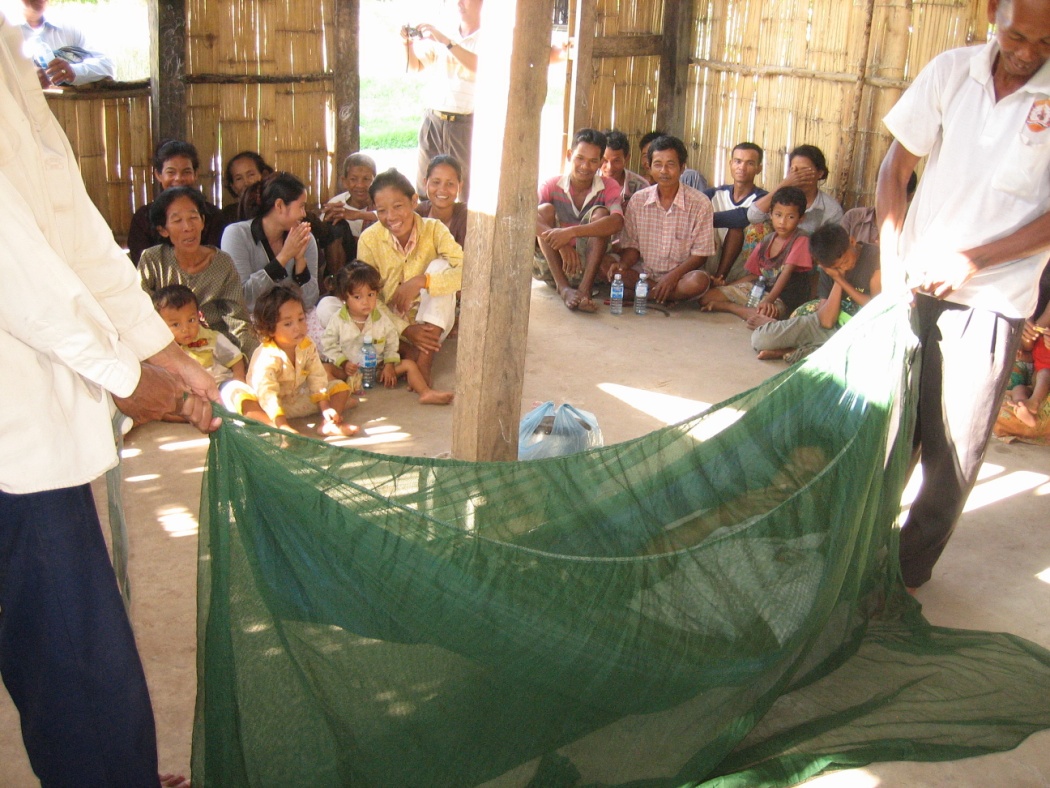


A further activity included one in which PD behaviours were written onto a card and placed in a cardboard box decorated as a PD house. Participants were asked to take out a piece of paper through the house door and read the behaviour. Each behaviour lead to a general discussion among the participants and, at the end, an action plan was prepared to conduct activities that could enable more families to adopt these behaviours.

3) Community handover seminar

Activities in the community handover seminar were designed to increase community participation and motivation to conduct PD behaviours. They included:

- Illustration competition: The competition was organized at the community level by the PD volunteers one week ahead of the seminar to generate interest in the seminar and reinforce key PD malaria messages in the communities. The PD volunteers selected the 10 best illustrations/sketches related to malaria control and prevention from the target communities. These were displayed at the seminar venue, and the audience reviewed the messages and pictures and selected the three best illustrations for prizes.
- Malaria song competition: Prior to the seminar, PD volunteers identified two or three students, teachers or community members from each target community/village for the competition. The participants prepared and sang malaria songs to reinforced key messages from the PD malaria project. During the seminar, the audience selected the best song for a final prize.
- Quiz: Questions related to key malaria messages were written on small pieces of paper, folded and placed in a basket. The seminar audience was asked the questions one-by-one and upon giving the correct answer, an audience member received a small gift/souvenir from the project field team. The purpose of this segment was to reinforce messages and clarify different issues related with malaria prevention and treatment.
- Symbolic handover to the community: At the end of the seminar, the project was handed over to the PD volunteers and community members. The community members interacted and discussed to develop a future action plan to ensure continuation of the PD activities in the target communities.
